# Exploring the Plant Growth-Promotion of Four *Streptomyces* Strains from Rhizosphere Soil to Enhance Cucumber Growth and Yield

**DOI:** 10.3390/plants11233316

**Published:** 2022-12-01

**Authors:** Ayman F. Omar, Adil H. A. Abdelmageed, Ahmad Al-Turki, Noha M. Abdelhameid, R. Z. Sayyed, Medhat Rehan

**Affiliations:** 1 Department of Plant Production and Protection, College of Agriculture and Veterinary Medicine, Qassim University, Buraydah 51452, Saudi Arabia; 2 Plant Pathology and Biotechnology Lab, EPCRS Excellence Center, Department of Plant Pathology, Faculty of Agriculture, Kafrelsheikh University, Kafrelsheikh 33516, Egypt; 3 Department of Horticulture, University of Khartoum, Shambat, Khartoum North 13314, Sudan; 4 Desert Research Center (DRC), Soil Fertility and Microbiology Department, Cairo 11753, Egypt; 5 PSGVP Mandal’s S I Patil Arts, G B Patel Science & STKVS Commerce College, Shahada 425409, India; 6 Department of Genetics, Faculty of Agriculture, Kafrelsheikh University, Kafr El-Sheikh 33516, Egypt

**Keywords:** cucumber, IAA, phosphate solubilization, plant growth promoting bacteria (PGPB), siderophore, *Streptomyces*

## Abstract

The genus *Streptomyces* is the most abundant and essential microbes in the soil microbial community. *Streptomyces* are familiar and have great potential to produce a large variety of bioactive compounds. This genus considers an efficient biofertilizer based on its plant growth-promoting activities. Based on their ability to produce a wide varieties of bioactive molecules, the present study aimed to explore the potential plant growth promotion of four *Streptomyces* strains and their role in enhancing cucumber growth and yield under greenhouse conditions. *Streptomyces* sp. strain HM2, *Streptomyces thinghirensis* strain HM3, *Streptomyces* sp. strain HM8, and *Streptomyces tricolor* strain HM10 were chosen for the current study. Plant growth-promoting (PGP) features, i.e., indole acetic acid (IAA) production, siderophore excretion, and solubilizing phosphate, were evaluated in vitro. All four strains produced IAA, siderophore, and immobilized inorganic phosphate. Following 4 days of incubation at 30 °C, strains HM2, HM3, HM8, and HM10 produced copious amounts of IAA (18, 22, 62, and 146 µg/mL, respectively) and siderophores (42.59, 40.01, 16.84, 64.14% SU, respectively). At the same time, P solubilization efficacy scored 64.3%, 84.4%, 57.2%, and 81.6% with the same frequency. During in planta evaluation, selected *Streptomyces* strains combined with rock phosphate were assessed as biofertilizers on the growth and yield of cucumber plants. Under all treatments, positive and significant differences in studied traits were manifested except dry stem matter (SDM), net assimilation rate (NAR), relative growth rate (RGR), and fruit firmness (FF). Treatment T4 (rock phosphate + strain HM3) followed by T5 (rock phosphate + strain HM8) revealed the best results for plant height (PH), number of leaves per plant (NLPP), root length (RL), number of fruits per plant (NFPP), fruit length (FL), fruit diameter (FD), fruit fresh weight per plant (FFWPP), soil P (SP) after 21 DAT, and soil P at the end of the experiment. Notably, T6 (rock phosphate + strain HM10) caused a considerable increase in leaf area (LA). Plant growth-promoting bacteria enhance plant growth and yield through phosphorus solubilizing, improve nutrient availability, produce phytohormones, and support plant growth under abiotic stress. These features are important for sustainable agriculture and reducing environmental pollution with chemical fertilizers and pesticides.

## 1. Introduction

The rhizosphere area is the narrow region of soil around the plant roots directly influenced by root secretions and microbial associations. The rhizosphere harbors beneficial microorganisms known as plant growth-promoting bacteria (PGPB) [1,2]. These PGPB have been known as biofertilizers in modern agriculture and as a new tool to enhance crop growth and productivity in sustainable agriculture [2]. PGPB colonizes the rhizosphere and facilitates plant growth directly by stimulating the growth or indirectly via biocontrol of the phytopathogens. The direct plant growth stimulation activity is through increased availability of nutrients, such as synthesis of phytohormones (i.e., auxin, cytokinins, gibberellins, abscisic acid) [3], bio-fixation of atmospheric nitrogen [4], production of siderophores (iron sequestration) [5], solubilization of soil minerals (i.e., phosphorus and potassium) [6,7], ACC (1-Aminocyclopropane-1-Carboxylate) deaminase activity [8], and in various other ways [9,10,11,12]. The indirect mechanisms involve biocontrol of phytopathogens through antibacterial and antifungal biomolecules [13], development of resistance against phytopathogens (i.e., soil-borne disease) [14,15,16,17], and competition for nutrients [13,14,15,16,17,18,19,20,21]. In addition, PGPB mitigates various abiotic and oxidative stresses [18,19,20,21,22,23,24,25,26,27,28,29,30]. Their use reduces chemical inputs in agriculture and environmental pollution [31,32].

Actinobacteria represent a significant microbial population in the soil, particularly in the rhizosphere [33]. They are a broad group of filamentous, Gram-positive, spore-forming, aerobic bacteria that play an essential role in soil nutrient recycling and ecology [34,35,36,37]. *Streptomyces* genus is the most broadly studied bacteria and is a rich source of a wide variety of biologically active compounds such as antibiotics, antiparasitic, antimicrobial, and immune-suppressing [38,39,40,41]. In addition to being a biocontrol agent, *Streptomyces* exhibits many traits that promote plant growth by producing the phytohormone indole acetic acid (IAA), fixing the atmospheric nitrogen, and solubilizing phosphates [34,35,36,37,42].

Production of IAA and phosphate solubility by *Streptomyces* sp. and their effects on *Arabidopsis* and *Brassica* sp. were previously assessed. *Streptomyces* sp. produced moderate levels of IAA and solubilized phosphate that significantly increased *Arabidopsis* and *Brassica* sp. vegetative growth and enhanced tolerance to abiotic stress [36]. The production of IAA, extracellular enzymes, siderophores, P solubilizing ability, and antifungal and antibacterial antagonism were demonstrated in rice by three strains of *Streptomyces*. For example, *Streptomyces fradiae* NKZ-259 produced IAA up to 82.36 μg/mL in the presence of tryptophan (2 g/L) after incubation for 6 days. This production from IAA significantly increased root and shoot lengths up to 7.97 and 32.77 cm, respectively, in tomato seedlings [42].

The cucumber, *Cucumis sativus* L., is one of the most popular members of the *cucurbitaceae* family. It is a tender annual vegetable vine crop grown for its fresh fruit in salads or taken as fresh fruit desserts [43]. It is well known as a natural diuretic and thus can serve as an active drug for secreting and promoting the flow of urine. The cucumber crop is considered one of the vegetable crops stressing the soil, which needs a high percentage of chemical fertilizer to obtain good growth and high production [44]. It is one of the main greenhouse vegetable crops widely grown in Saudi Arabia. The total greenhouse area for cucumber production in 2020 was 1679.7 hectares, producing 172,036.7 tons [45,46].

Chemical fertilizers are used too often to promote soil fertility and crop yield. Therefore, organic agriculture has attracted more attention in recent years. Organic farming is becoming of interest because of the perceived health and environmental benefits of reducing the number of toxic compounds such as nitrates [47,48]. The advantage of adding bacteria to the soil is secreting phytohormones, and organic acids, which change the pH and facilitate the micro-elements such as iron, zinc, manganese, boron, copper, and phosphorous [49].

Dursun et al. [50] and Isfahani and Besharati [51] observed a significant increment in length, fresh and dry weight of roots and shoots, and yield of inoculated cucumber plants when grown in soils treated with or without chemical fertilizers in a field environment. In a follow-up study, Isfahani et al. [52] reported a significant effect of *Pseudomonas* sp. and PSB, such as *P. putida* and *P. agglomerans* and chemical fertilizers, on the growth and yield components of cucumber.

Several PGPB can be used for future research to improve the health and quality of vegetables grown in different production systems. The present study focused on using multipotent *Streptomyces* as biofertilizers, assessing their production from IAA, siderophore, and phosphate solubilization, and their possible beneficial effects on the growth and yield of cucumber crop under greenhouse application in Qassim, Saudi Arabia.

## 2. Results

### 2.1. In Vitro Evaluation of Plant Growth Promotion Features

**Figure 1:**
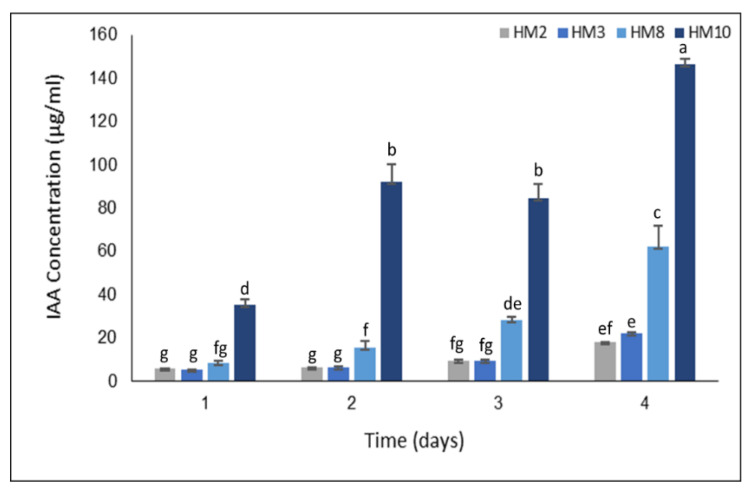
IAA production by four *Streptomyces* strains (HM2, HM3, HM8, and HM10) in King’s medium B supplemented with 0.2% L-tryptophan over 4 days, each value representing the mean of three replicates. Bars not sharing similar letters are significantly different (*p* > 0.05).

Four *Streptomyces* strains (*Streptomyces* sp. strain HM2, *Streptomyces thinghirensis* strain HM3, *Streptomyces* sp. strain HM8, and *Streptomyces tricolor* strain HM10) were studied in the current investigation for their potential PGP activity. In vitro evaluation of IAA production by selected strains revealed high production from IAA after 96 h of incubation. Strain HM2 produced up to 18 µg/mL when incubated for 4 days, whereas strain HM3 displayed 22 µg/mL. Strains HM8 and HM10 were higher in their IAA production, with productivity increasing gradually and reaching 62 and 146 µg/mL, respectively, as a response to 0.2% from L-tryptophan (Figure 1).

**Figure 2:**
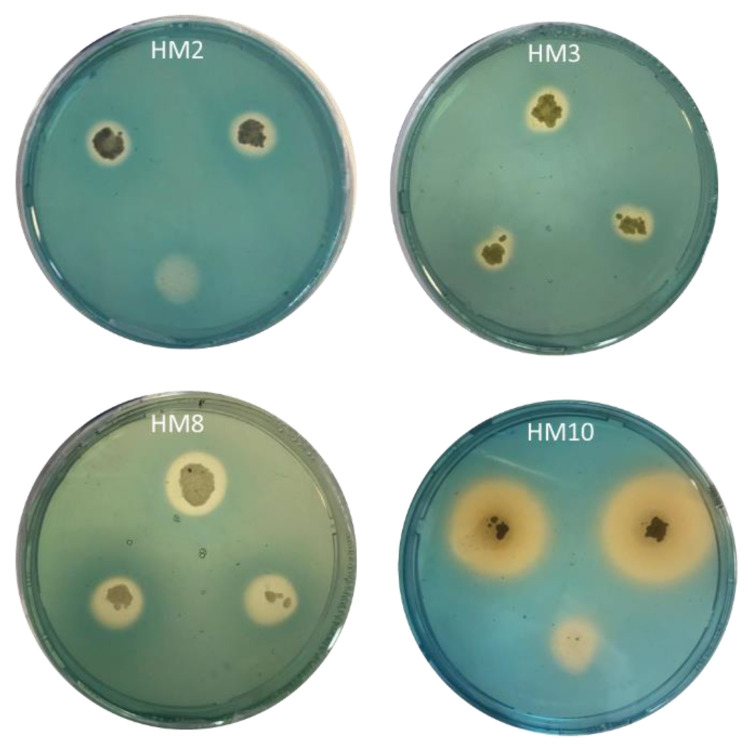
Siderophore production by the four *Streptomyces* strains detected in CAS--King’s blue agar medium. The orange-yellow halo around colonies represents siderophore production.

**Figure 3:**
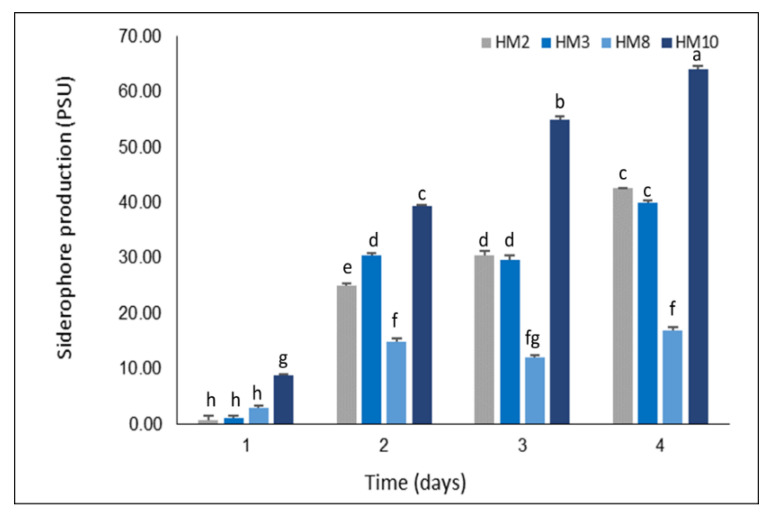
Represents the quantified production of siderophore by the four *Streptomyces* strains in King’s broth medium. Bars not sharing similar letters are significantly different (*p* > 0.05).

Siderophore production was assayed on solid and liquid CAS--King’s blue agar media. The four tested strains displayed a pattern of siderophore production with an orange-yellow faint halo around the colonies on the CAS medium (Figure 2). *S. tricolor* HM10 secreted more siderophore with a bigger halo around colonies in a short growth time compared to other strains. When quantifying the production in broth cultures, strain HM10 excreted a high siderophore amount, reaching 64.14 psu, followed by strains HM2 and HM3 with 42.6 and 40 psu, respectively (Figure 3).

**Figure 4:**
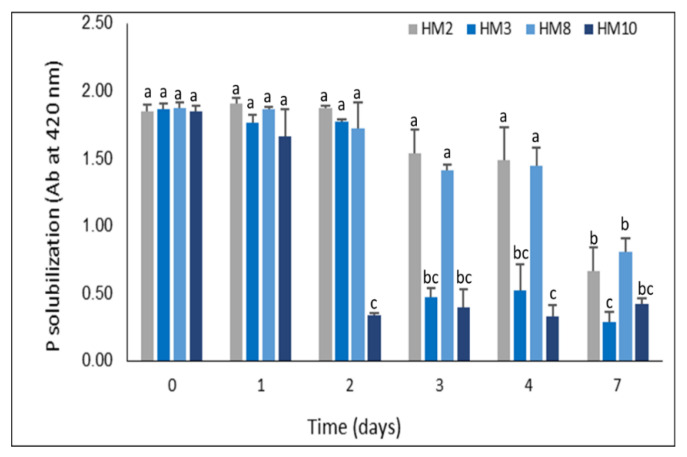
Phosphate solubilization of inorganic phosphate by the selected four *Streptomyces* strains grown in NBRIP-liquid medium supplemented with 5 g/L of Ca_3_(PO_4_)_2_ for 7 days, each value represents the mean of three replicates. Bars not sharing similar letters are significantly different (*p* > 0.05).

Furthermore, *Streptomyces* strains were tested for their ability to solubilize inorganic phosphate. The P solubilization activity was evaluated in both NBRIP-agar/broth media supplemented with tricalcium phosphate (Ca_3_(PO_4_)_2_). All designated strains exhibited P solubilization activity in the broth medium, reaching the maximum after 168 h. *S. thinghirensis* strain HM3 showed the maximum P solubilization with 84.4% efficiency from the beginning amount of inorganic phosphate. Moreover, *S. tricolor* HM10 could solubilize P with efficacy reached 81.6% after 24 h, followed by strains HM2 and HM8 with capability measured at 64.3% and 57.2%, respectively (Figure 4).

**Figure 5:**
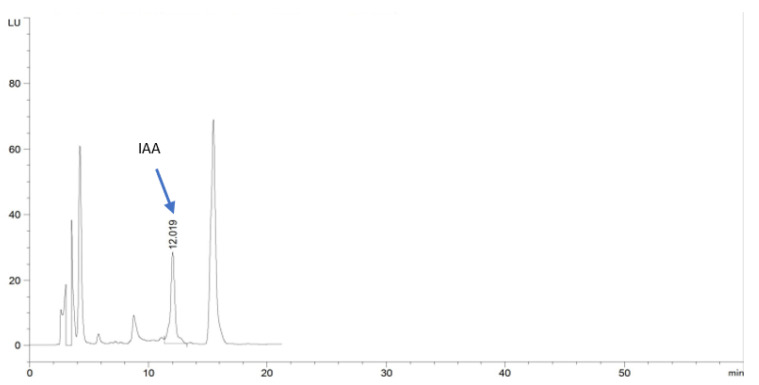
Analysis of produced IAA production by *S. tricolor* HM10 using HPLC.

When subjected to the extracted and purified *S. tricolor* HM10 broth into HPLC, a reliable peak with a retention time of 12.019 min was detected in the bacterial broth. The detectable peak was comparable to this of the authentic IAA standard. The obtained peak confirmed that the HM10 strain produced IAA and subsequently belongs to PGPB (Figure 5).

### 2.2. Streptomyces Strains and Their Effect on Plant Growth Promotion in Cucumber Plants

#### 2.2.1. Growth Traits

Vegetative growth traits such as plant length (cm), number of leaves per plant, Root length (cm), Leaf DM (dry matter) %, Stem DM%, and Root DM% are shown in Table 1. The assessed traits manifested a wide variation for all measured traits under treatment conditions.

Fertilization treatments T4 (rock phosphate (RP) + strain HM3) gave the highest and most significant values for plant height (198.33 cm) as well as T5 (RP + strain HM8) and T6 (RP + HM10), with their number of leaves plant^−1^ (26.66) and root length (44.66). Meanwhile, T2 (RP) gave the lowest significant values in plant height (124.00 cm), the number of leaves plant^−1^ (16.66), and root length (17.00) (Table 1).

Regarding leaf dry matter percentage, the highest value was obtained by treatment T2 (RP), and the lowest value was obtained by treatment T3 (RP + Strain HM2). Treatment T2 gave the highest value for stem dry matter percentage, while treatment T1 (NP) produced the lowest value of this trait without any significant differences between treatments. T7 treatment (RP + mixed strains) gave the highest positive and significant value of root dry matter as a percentage, whereas T3 (RP + strain HM2) recorded the lowest number in this trait.

Leaf area (cm^2^), net assimilation rate (g m^−2^·day^−1^), and relative growth rate (g·g^−1^·day^−1^) are located in Table 2. Treatments T6 (RP + strain HM10) and T5 (RP + strain HM8) exhibited the highest value for leaf area (323.20 and 323.00 cm^2^), respectively, while the treatments T1 (NP) and T2 (RP) displayed the lowest and most significant values (188.16 and 210.067 cm^2^) for this trait (Table 3). Concerning net assimilation rate (NAR) and relative growth rate (RGR), there are no significant differences among all treatments, recording the highest values for net assimilation rate and relative growth rate (0.008 and 0.137), respectively, for treatments T1 and T5. On the other hand, in treatments T2, T6, and T7, the net assimilation rate declined to 0.006 g cm^−2^·day^−1,^ and treatments T1, T3, and T7 showed the lowest values for relative growth rate (0.117 g g^−1^·day^−1^), respectively.

#### 2.2.2. Yield Traits Measurements

Fruit characteristics such as fruit length (cm), fruit diameter (cm), number of fruits plant^−1^, fruit fresh weight (g), and fruit firmness are presented in Table 3. A significant difference between treatments was detected in all fruit yield characteristics except fruit firmness. T4 treatment recorded a considerable increase in all studied fruit characteristics except fruit firmness. Otherwise, the lowest values in all studied characteristics except fruit firmness were given by the treatment T2 under adding rock phosphate.

#### 2.2.3. Soil and Plant Phosphorus

A combination of *Streptomyces* and RP fertilization significantly affected rhizospheric soil phosphorus concentrations compared with control T2 (RP only). The four tested *Streptomyces* strains showed different abilities to release soluble phosphate from RP (Table 4). The highly available P was noticed in T4 (RP + strain HM3), 30.76 and 26.36 mg/kg, followed by T5 (RP + strain HM8), 27.76 and 22.40 mg/kg, at 21 DAP and at harvest, respectively. In contrast, the T2 (RP) had the lowest available P concentration, 13.90 mg/kg at harvest. The maximum significant and positive plant P was observed in treatment T4 (0.91%) followed by treatment T5 (0.75%), whereas no significant differences were found between the other treatments.

#### 2.2.4. Interrelationship among Traits

**Figure 6:**
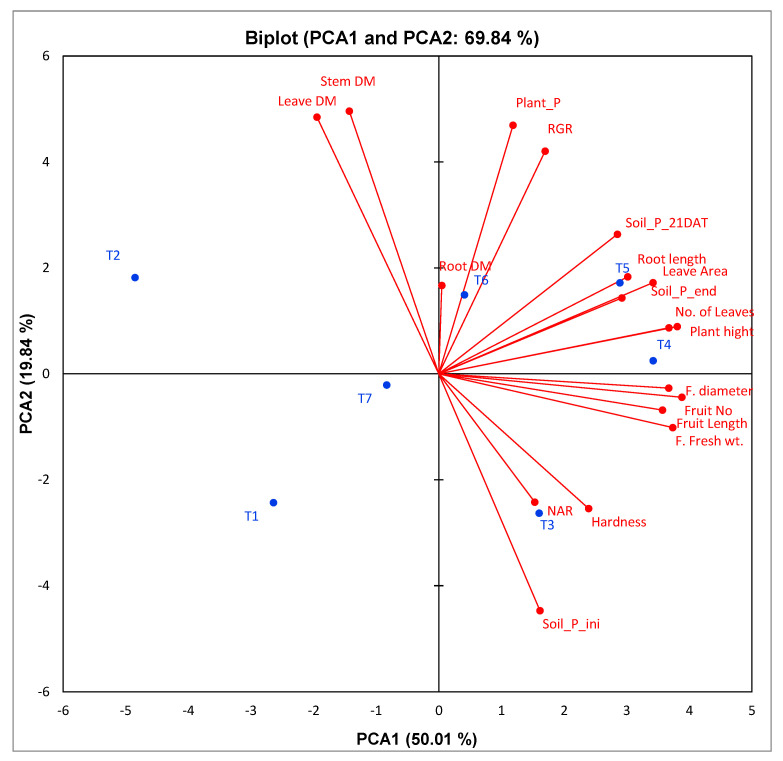
PC-biplot exploring the relationship between the evaluated traits under treatments of rock phosphate and *Streptomyces* strains as plant growth-promoting agents.

To assess the relationship between the evaluated traits, principal component analysis was achieved to categorize the related growth and yield traits together under the action of applied plant growth-promoting bacteria. The first two PCAs represented 69.84% (50.01% for PCA1 and 19.84% for PCA2) of the trait variability. Consequently, the two PCAs were utilized to draw the PC-biplot (Figure 6). A strong positive correlation was among plant content of P, relative growth rate (RGR), soil P after 21 days from planting, leaf area, root length, soil P at the end of planting, plant height, number of leaves, fresh weight of fruit, fruit number, fruit diameter, and fruit length. Furthermore, T3, T4, T5, and T6 are on the positive side of PCA1, along with most growth and yield traits.

**Figure 7:**
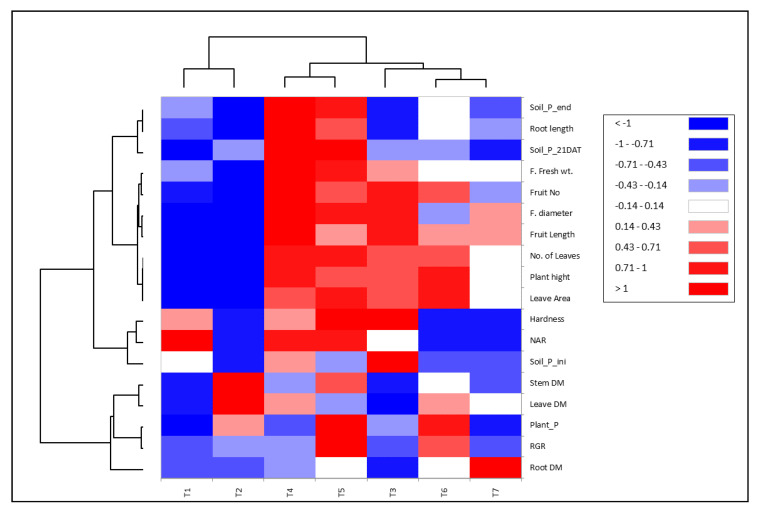
Heatmap correlation between evaluated traits and appropriate treatments from *Streptomyces* strains with rock phosphate.

To explore the relationship between quantified traits and applicable treatments of *Streptomyces* strains, heat map analysis was employed to disclose this interaction (Figure 7). The applied treatments were categorized into two groups, one group involved T1 and T2 (non-treated plants with *Streptomyces* strains), whereas the second group revealed two clusters. One cluster contained T4 and T5 treatments, while the second cluster implied the rest of the treatments with *Streptomyces* (T3, T6, and T7). Interestingly, application of *Streptomyces* strains along with rock phosphate (T4 and T5) exhibited positive and significant correlation with growth traits, i.e., number of leaves, plant height, leaf area, soil P at the end, soil P after 21 days from planting, root length relative growth rate, fruit fresh weight, fruit number, fruit diameter, fruit length, and hardness. Moreover, T3 followed by T6 displayed a positive correlation with most previous traits except soil P at 21 days and at the end, root length, and net assimilation rate (NAR).

## 3. Discussion

IAA production is considered vital for plant growth. Plant growth-promoting bacteria improve crop yield and thus reduce yield costs [11]. *Streptomycetes* can stimulate and enhance plant growth by producing phytohormones, solubilizing inorganic phosphate, and biosynthesis of siderophore that chelating iron from the environment and inhibit the phytopathogens [34,35,53]. As one of the soil’s most abundant microbes, four isolated *Streptomyces* strains from Qassim, SA, were evaluated in vitro as plant growth-promoting bacteria. The results indicated that selected *Streptomyces* strains produced IAA with different levels since strain HM2 recorded the least amount (18 µg/mL), whereas HM10 secreted the highest amount (146 µg/mL) after 4 days of incubation at 30 °C (Figure 1). Dimkpa et al. [54] described that *Pseudomonas chlororaphis* O6 produces about 30 μg/mL of IAA under the sub-lethal levels of CuO and ZnO nanoparticles in a medium containing tryptophan as a precursor. Moreover, *Bacillus megaterium* ST2-9, isolated from soil salt samples in the delta of Vietnam, produced 33.13 mg/L of IAA after 8 days of incubation [55]. Notably, Lebrazi et al. [56] isolated *Rhizobium* sp. strain I69 while production from IAA reached 135 μg/mL, increasing to 166 μg/mL with optimal parameters, 36 °C incubation, 6.5 pH, 1 g/L tryptophan and 0.1 g/L NaCl. *Streptomyces fradiae* NKZ-259 [38] and *Streptomyces hydrogenans* DH16 [57] achieved the maximum production from IAA (82.363 and 80.06 μg/mL) in the presence of tryptophan. Moreover, three *Streptomyces* strains identified as *Streptomyces kunmingenesis* WC-3, *Streptomyces nobilis* WA-3, and *Streptomyces enissocaesilis* TA-3 produced 79.23, 79.5, and 69.26 μg/mL, respectively, of IAA as a response to 0.5 mg/mL of L-tryptophan [58].

*Streptomyces* among soil microbes is recognized with siderophores production. Microbes can bind Fe3+ from the environment by producing a siderophore and making it available for its growth, and plants can also utilize these as an iron source [5]. Regarding siderophore production, our tested four strains (HM2, HM3, HM8, and HM10) disclosed siderophore production on CAS-King’s agar media by forming a clear orange halo zone around the colonies (Figure 2). Quantifying the siderophore production as psu by CAS solution reflected high production by strain HM10 exceeding 64.14 psu (Figure 3). This strain secretes three siderophore types (catechol, hydroxamate, and carboxylate) [59]. Similarly, Anwar et al. [58] isolated 98 rhizospheric actinomycetes from wheat and tomato fields. All rhizobacterial isolates recorded positive attitudes in siderophore, hydrogen cyanide, and ammonia production.

In phosphate solubilization, all four strains possess phosphate solubilization in different efficiency. *S. thinghirensis* HM3 released the highest amount from inorganic phosphate with an efficiency of 84.4% after 7 days of incubation (Figure 4). Six *Streptomyces* could solubilize phosphate after 7 days of incubation by producing clear zones around the colonies. Based on the solubilization index, *Streptomyces* sp. WA-1 and *S. djakartensis* TB-4 could solubilize 72.13 and 70.36 mg/100 mL, respectively [54]. Chouyia et al. [60] isolated and characterized *Streptomyces roseocinereus* and *Streptomyces natalensis* with a phosphate solubilization index of 1.75 and 1.63, respectively. Remarkably, *Streptomyces* sp. CoT10 mobilized different P resources, particularly 72.49 mg/L of FePO_4_, consequently improving phosphate content in soil by 15.35%, which supplied plant growth with high P and Fe acquisition [61]. On the same trend, *Pantoea* sp. S32 isolated from alfalfa rhizosphere, immobilized P with efficacy exceeded 18.38, 3.07, 0.16, 0.51, and 2.62% in cultured media containing CaHPO_4_, lecithin, rock phosphate, FePO_4_, and AlPO_4_, respectively [58]. On a closer look, phosphate-dissolving bacteria can solubilize phosphate in two ways: (1) by secreting organic acid that dissolves inorganic P compounds, and (2) by producing phosphatase enzymes that mineralize P compounds [62,63].

Various reports have demonstrated a significant increment in the vegetative growth traits when investigating the effect of organic and biofertilizers on the growth and development of different crops [23,64,65,66,67,68,69,70,71,72,73,74]. In the present study, the results revealed that treatments T4 (rock phosphate (RP) + strain HM3) indicated the highest and most positive significant values for plant height, number of leaves per plant, and root length (Table 1). Furthermore, leaf dry matter and stem dry matter percentages are applied by treatment T2 (RP). Otherwise, T7 (RP + mixed strains) was recognized as the highest positive and significant value of root dry matter. The results of the present study are inconsistent with that of Eifediyi, and Remison [63], who stated that increasing the rates of inorganic fertilizers leads to increased growth in the cucumber crop.

The reason for increasing the vegetative growth traits when treated with rock phosphate or rock phosphate with biofertilizers may be attributed to increasing soil fertility and thus increasing the absorption of nutrients such as phosphorous, potassium, and others by the plant, which have a significant role in many vital processes leading to the division of cells forming meristem tissues and increase the size of cells. Moreover, other substances such as hormones, vitamins, proteins, and some amino acids such as alanine and glycine enhance the growth rate. These biofertilizers also improve the structure and porosity of the soil, which leads to a balance between moisture and aeration in the soil [75,76].

Ashraf et al. [77] disclosed that IAA application in barley revealed effective enhancement in growth and photosynthetic efficiency under normal and water deficit conditions. The significant and positive effects for leaf area and relative growth rate were detected under treatments T5 and T6. Consequently, adding IAA significantly increased the number of leaves/plant, leaf area, leaf area index, total dry matter, plant height, number of branches/plant, and crop growth rate in mungbean (*Vigna radiata* L.) [78,79]. These increments may be due to the benefits of high IAA production by strains HM8 and HM10 (62 and 146 µg/mL).

The results of yield traits, including fruit length, fruit diameter, number of fruits plant^−1^, and fruit fresh weight, elucidated the significant difference between treatments in all fruit yield characteristics except fruit firmness. T4 showed the best considerable increase in all studied fruit characteristics except fruit firmness (Table 3). These data coincide with those obtained by Mahmoud et al. [80], Nair and Ngoajio [81], Isfahani and Besharati [51], Abdrabbo and Desoky [82], and Tahir et al. [83]. They demonstrated a significant increment in most fruit yield characteristics when different types of organic and biofertilizers were added separately or in combination with other crops.

Concerning the fruit firmness (Lbs. inch^−2^), there are no significant differences among the treatments (Table 4). Despite that, the treatments T3 and T5 performed the highest value (5.00 Lbs. inch^−2^). Results coming from our study are in harmony with those disclosed by Azarmi et al. [79]; Zainuddin [69]; Valdenegro et al. [84]; Kang et al. [85]; and Zapata-Sifuentes et al. [82], who depicted a significant increase in the fruit quality parameters, i.e., fruit diameter, fruit size, biomass, and yield, as well as antioxidant capacity.

Numerous *Streptomyces* spp. isolated from rhizosphere soils are known as superior phosphate solubilizers and plant growth promoters. The tested strains showed high phosphate solubilization and may consider P suppliers in soils rich with inorganic phosphate (Table 4). As previously documented by Jog et al., *Streptomyces* spp. has been reported as the best phosphate solubilizers [86] by Sadeghi et al. [87]; Jog et al. [88]; and Sreevidya et al. [89]. Plant growth-promoting bacteria help phosphorus solubilize, improve nutrient availability, control phytopathogens, produce phytohormones, biosynthesize metal chelators, and alleviate abiotic stress in plants [16,90,91,92,93,94]. These properties are considered an important practice for sustainable agriculture and good alternative practices to reduce environmental hazards with chemical fertilizers and pesticides. PGPR is safe for plants, the environment, and all living organisms. PCA is a technique that analyzes data tables with quantitative dependent variables to describe the inter-correlation between these data. Appling PCA and heatmap depicted the correlation between measured growth and yield traits under treatment of plants with different *Streptomyces* strains as plant growth-promoting agents (Figure 6 and Figure 7). A positive and significant correlation was observed between plant P, relative growth rate (RGR), soil P level, leaf area, root length, plant height, number of leaves, fruit fresh weight, fruit number, fruit diameter, and length when inoculating with *Streptomyces*. Moreover, treatments T3, T4, T5, and T6 are presented on the positive side of PCA1, along with most growth and yield traits. Sherpa et al. [95] used PCA to correlate various physicochemical parameters and the microbial community of peas (*Pisum sativum* L.). Firmicutes of the bulk soil revealed a positive correlation to available potassium and soil organic carbon (SOC); furthermore, proteobacteria showed a high correlation with the pH and available phosphorus.

## 4. Materials and Methods

### 4.1. Lab Work

#### 4.1.1. *Streptomyces* Strains and Cultural Conditions

Four *Streptomyces* strains isolated from the Qassim region, Saudi Arabia, were chosen for the current study. *Streptomyces* sp. strain HM2 (accession, MN527230), *Streptomyces thinghirensis* strain HM3 (accession, MN527231), *Streptomyces* sp. strain HM8 (accession, MN527235) and *Streptomyces tricolor* strain HM10 (accession, MN527236) were isolated and identified as described previously by Rehan et. al. [96]. For regular growth and maintenance, Tryptic Soy Broth/agar (TSB/A), composed of (g/L) peptic digest of soybean meal 5.0, sodium chloride 5.0, agar 15.0, pH 7.5. was applied. Moreover, Glucose Soybean meal Agar (GSA), which consisted of (g/L), 10 soybean meal, 10 glucose, 1 CaCO_3_, 10 NaCl, and pH adjusted to 7.5, was used for spore production for 5 days at 30 °C.

#### 4.1.2. In Vitro Evaluation of IAA, Siderophore, and Phosphate Solubilization of *Streptomyces* Strains

Solid and liquid King’s medium B base containing 20 g peptone, 1.5 g K_2_HPO_4_, 1.5 g O_4_.7H_2_O, 15 g agar per liter, pH 7.5 were used for siderophore and IAA detection and production. Pure bacterial colonies were inoculated in liquid King’s B medium supplemented with 0.2% L-tryptophan in an incubator shaker (200 rpm) for 4 days at 30 °C, one milliliter from cultured bacteria was withdrawn each 24 h and kept at −20 °C. Indole acetic acid (IAA) was quantified following the method of Gang et al. [97] involving the Salkowaski reagent (0.5 M FeCl_3_ and 35% HClO_4_). One volume (100 µL) from cultures supernatant (after centrifugation at 10,000 rpm for 3 min) was mixed with an equal volume of Salkowaski reagent in a microplate reader, vortex gently, and incubated in a dark condition at 30 °C for 30 min. The mixture was subjected to color intensity measurement spectrophotometrically at 535 nm against an uninoculated medium and a standard curve generated by known concentration from IAA.

Siderophores detection and production were quantified by CAS assay (Chrome Azurol S) [98,99]. CAS solution was prepared by dissolving both: 72.9 mg (hexadecyl trimethyl ammonium bromide (HDTMA)) in 40 mL ddH_2_O, and 60.5 mg (chrome azurol S) in 50 mL dH_2_O, then mixed together and 10 mL from Fe^3+^ solution (1 mM FeCl_3_ in 10 mM HCl) was added. CAS solution was then autoclaved and mixed with autoclaved King’s medium with a 1:9 ratio, respectively, after cooling to 50 °C. Siderophore quantification was performed by mixing equal volumes (100 µL) from cultured supernatants and CAS reagents. This was followed by incubation at room temperature for 20 min and absorbance measured at 630 nm in a microplate reader [100]. Siderophore production was calculated as percent siderophore unit (psu) according to the method of Payne [101].

To detect phosphate solubilization, *Streptomyces* strains were grown in NBRIP medium containing (g/L), glucose, 10; MgCl_2_.6H_2_O, 5; MgSO_4_.7H_2_O, 0.25; KCl, 0.2; (NH_4_)_2_SO_4_, 0.1; Ca_3_(PO_4_)_2_, 5; agar 15; [102] and incubated at 30 °C for 5 days. Solubilized phosphate was detected by forming a clear zone in the culture plate. Liquid NBRIP medium was applied for phosphate solubilization rate by growing *Streptomyces* strains for 7 days with incubation shaking (30 °C and 200 rpm). Manullang and Chuang [36] described that the absorbance was measured in a microplate reader at 420 nm, and a decrease in absorption values determined phosphate solubilization.

#### 4.1.3. Extraction, Purification, and Determination of IAA by HPLC

*S. tricolor* HM10, the highest IAA producer, was grown in King’s B medium supplemented with 0.2% L-tryptophan for 72 h. The fermented broth was filtered using a Whatman No.1 filter paper, and 10 mL from the filtrate was mixed with an equal volume of acidified acetonitrile (0.1% acetic acid) and shaken for 2 min. Furthermore, 4g of MgSO_4_ and 1 g of sodium acetate, followed by shaking for 1 min, were added, then centrifugation at 10,000 rpm for 5 min.

The supernatant was filtrated through a 0.45 µm syringe filter, then diluted 2× with water for HPLC-FLD analysis with the following condition: 1220 infinity HPLC (Agilent, USA), fluorescence light detector (FLD) (excitation 282 nm, Emission 360 nm), Column (C8, 250 mm*4.6) (5 µm particle size) (zorbax-Agilent) Gradient elution consist of: (A) water (PH of 3.8), (B) acetonitrile, 0 min (A:B) (80%:20%), 25 min (A:B) (50%:50%), 31 min (A:B)(80%:20%), 40 min (A:B) (80%:20%). The injection volume was 20 µL, and the column temperature reached 30 °C.

### 4.2. Greenhouse Work

#### 4.2.1. Experimental Site and Agronomic Practices

This experiment was conducted at the experimental farm, College of Agriculture and Veterinary Medicine, Qassim University, Al-Qassim region, Kingdom of Saudi Arabia (latitude 26–27 N, longitude 44–45 E, altitude 725 m above sea level) to investigate the effect of four *Streptomyces* strains in the presence of rock phosphate as biofertilizers on the growth and yield of cucumber vegetable crop. Soil and water samples were collected before planting at 0–30 cm depth. The properties of soil samples and other details are given in Table 5.

The recommended dose of NPK as mineral fertilizers was applied as follows: 280 kg N/ha as 600 kg urea (46% N), 465 kg K_2_O/ha as 930 kg potassium sulfate (48% K20), and 195 kg P_2_O_5_/ha as 672 kg rock phosphate (29% P_2_O_5_) or 1050 kg calcium superphosphate (18.5% P_2_O_5_) as control treatment. Before transplanting, all quantities of calcium superphosphate and rock phosphate were added to cucumber pots, while urea and potassium sulfate were added in four equal portions before transplanting, then after 2, 4, and 6 weeks.

#### 4.2.2. *Streptomyces* Treatments under Greenhouse

The cultures were prepared by growing the four *Streptomyces* strains in King’s B medium with shaking for 72 h at 30 °C. The fermented broth was homogenized with a syringe, and glucose (5 g/100 mL) was added as the primary carbon source when inoculating the seedlings. At the time of seedlings transplanting, inoculum from each strain and mixture from all strains was added around the seedlings’ roots (approximately 50 mL per plant). The inoculation process was repeated again after 15 days to ensure the presence of the bacterium around the roots.

There were seven fertilization treatments, namely, normal phosphate as control (T1), rock phosphate (RP) (T2), RP + strain HM3 (T3), RP + strain HM3 (T4), RP + strain HM8 (T5), RP + strain HM10 (T6), and RP + mixture of the four strains (T7). Phosphorus treatments were added to the soil before planting at a rate of 4.8 g of normal phosphate and 7.5 g of rock phosphate pot^−1^ (30 cm long by 30 cm diameter filled with 20.7 kg of sandy soil).

XTREME cucumber hybrid variety was selected for the current study. The seedlings of this cultivar were purchased from the Saudi United Fertilizers Company. After one month, seedlings were transplanted to the pots in the greenhouse at 50 cm apart and 1 m wide of the ridge. The experiment design was laid out in a completely randomized block design with 12 replicates for each treatment.

Average day and night temperatures in greenhouses were 25 °C and 18 °C, as Maynard and Hochmuth [103] described as the optimum temperatures for cucumber growth.

#### 4.2.3. Measured Traits

##### Morphological and Growth Traits

After 21 days of planting, three plants from each treatment were randomly selected for growth traits measurement, including; plant height (PH), number of leaves per plant (NLPP), root length (RL), leaf dry matter (Leaf DM), stem dry matter (Stem DM), Root DM that measured after 3 days of drying at 70 °C, and the leaf area (LA) according to Cho et al. [106] and Ding et al. [107].

The relative growth rate (RGR) (g·g^−1^·day^−1^), according to Evans [70] and Hunt et al. [20], was recorded using the following equation:

$$\mathrm{RGR}\;=\frac{{\mathrm{Ln}\;W}_{2}-{\;\mathrm{Ln}\;W}_{1}}{T_{2}-{\;T}_{1}}(g\cdot g^{-1}\cdot {\mathrm{day}}^{-1})$$

where: Ln: logarithm of the natural base, W_2_: dry weight of the plant at the end of the period T_2_, W_1_: dry weight of the plant at the beginning of the period T_1_, and W_1_ and W_2_ are the total DW (g/plant).

Net assimilation rate (NAR) (g m^−2^·day^−1^) was calculated using the following equation according to Evans [108] and Hunt et al. [46].

$$\mathrm{NAR}=\frac{W_{2}-W_{1}}{T_{2}-{\;T}_{1}}\times \frac{{\mathrm{Ln}\;\mathrm{LA}}_{2}-\mathrm{Ln}\;\mathrm{LA}}{{\mathrm{LA}}_{2}-{\;\mathrm{LA}}_{1}}(g\cdot m^{-2}\cdot {\mathrm{day}}^{-1})$$

where: W_2_: Dry weight of the plant at the beginning of the period T_2_, W_1_: dry weight of the plant at the beginning of the period T_1_, LA_2_: leaf area of the plant at the beginning of the period T_2_, LA_1_: leaf area of the plant at the beginning of the period T_1_, and Ln: logarithm of the natural base.

Leaf dry matter percentage (LDM%), stem dry matter percentage (SDM), and root dry matter percentage (RDM%) were determined 21 days after seeding. At the same date, three plants per treatment were randomly chosen for P measurement. Total phosphorous and concentrations were determined in soil and the dry matter of the fourth upper leaves, according to Cottenie et al. [109].

##### Grain Yield and Its Related Traits

Fruit length (FL), fruit diameter (FD), number of fruits per plant (NFPP), fruit fresh weight per plant (FFWPP) at the horticultural maturity stage, and fruit firmness (FF) were recorded regularly.

##### Statistical Analysis and Principal Component Analysis

Data were statistically analyzed, and Duncan’s test was used to compare the means at the significant level (*p* ≤ 0.05) according to Khiddir et al. [110] and Webster [111]. The normality of variables was verified using the Shapiro--Wilk test before proceeding with principal component analysis (PCA), and the correlation between different variables was measured by Pearson correlation. Additionally, Bartlett’s sphericity test and Kaiser--Meyer--Olkin (KMO) were performed. The PCA and Heat Map (HM) was performed using XLSTAT software version 2019. XLSTAT software version 2019 was used to classify the data into different clusters having a common trait [112] and was executed to define plant properties versus treatment groups.

## 5. Conclusions

The *Streptomyces* genus is familiar with producing bioactive molecules that can be used in medicine, pharmacy, and agriculture. Four isolated *Streptomyces* strains (*Streptomyces* sp. strain HM2, *Streptomyces thinghirensis* strain HM3, *Streptomyces* sp. strain HM8, and *Streptomyces tricolor* strain HM10) were tested for their potential plant growth promotion with cucumber under greenhouse conditions. The four strains produced IAA (18, 22, 62, and 146 µg/mL), siderophore (42.59, 40.01, 16.84, 64.14 %SU), and immobilized inorganic phosphate (64.3%, 84.4%, 57.2%, and 81.6%), respectively. Strain HM3, followed by strain HM8 and strain HM10, revealed the best results for plant height (PH), number of leaves per plant (NLPP), root length (RL), number of fruits per plant (NFPP), fruit length (FL), fruit diameter (FD), fruit fresh weight per plant (FFWPP), soil P (SP) after 21 DAT, and soil P at the end of the experiment.

## Figures and Tables

**Table 1:** Effect of chemical, organic, and biofertilizer treatments on some vegetative growth traits of cucumber plants grown under greenhouse conditions.

Treatments	Plant Height (cm)	No. of Leaves Plant^−1^	Root Length (cm)	Leaf DM (%)	Stem DM (%)	Root DM (%)
T1	133.00 ^c^	17.33 ^c^	21.33 ^bc^	13.55 ^b^	5.57 ^a^	5.84 ^ab^
T2	124.00 ^c^	16.67 ^c^	17.00 ^c^	16.11 ^a^	6.42 ^a^	6.21 ^ab^
T3	187.33 ^ab^	25.00 ^ab^	19.33 ^c^	13.08 ^b^	5.60 ^a^	5.32 ^b^
T4	198.33 ^a^	26.67 ^a^	44.67 ^a^	14.60 ^ab^	5.79 ^a^	6.67 ^ab^
T5	195.33 ^a^	26.33 ^a^	32.67 ^b^	14.01 ^ab^	6.02 ^a^	7.21 ^ab^
T6	196.33 ^a^	25.00 ^ab^	27.67 ^bc^	14.74 ^ab^	5.85 ^a^	7.34 ^ab^
T7	177.33 ^b^	22.67 ^b^	25.33 ^bc^	14.48 ^ab^	5.63 ^a^	11.10 ^a^

Values followed by the same letter (s) within each column did not significantly differ according to the Duncan multiple comparison test at the 5% level. Each value is the average of 3 replicates. ± Values indicated to standard error (±SE). Normal phosphate (T1), rock phosphate (RP) (T2), RP + strain HM3 (T3), RP + strain HM3 (T4), RP + strain HM8 (T5), RP + strain HM10 (T6) and RP + mixture of the four strains (T7). (DM) dry matter. ^a,b,c^: Values not sharing similar letters are significantly different (*p* > 0.05).

**Table 2:** Effect of chemical, organic, and biofertilizer treatments on some vegetative growth traits of cucumber plants grown under greenhouse conditions.

Treatments	Leaf Area (LA) (cm^2^)	Net Assimilation Rate (NAR) (g·cm^2^·day^−1^)	Relative Growth Rate (RGR) (g·g^−1^·day^−1^)
T1	188.167 ^c^	0.008 ^a^	0.117 ^a^
T2	210.067 ^c^	0.006 ^a^	0.120 ^a^
T3	307.333 ^ab^	0.007 ^a^	0.117 ^a^
T4	303.200 ^ab^	0.007 ^a^	0.120 ^a^
T5	323.000 ^a^	0.007 ^a^	0.137 ^a^
T6	323.200 ^a^	0.006 ^a^	0.127 ^a^
T7	267.933 ^b^	0.006 ^a^	0.117 ^a^

Values followed by the same letter (s) within each column did not significantly differ according to the Duncan multiple comparison test at the 5% level. Each value is the average of 3 replicates. ± Values indicated to standard error (±SE). Normal phosphate (T1), rock phosphate (RP) (T2), RP + strain HM3 (T3), RP + strain HM3 (T4), RP + strain HM8 (T5), RP + strain HM10 (T6) and RP + mixture of the four strains (T7). ^a,b,c^: Values not sharing similar letters are significantly different (*p* > 0.05).

**Table 3:** Effect of chemical, organic, and biofertilizer treatments on some fruit growth characteristics of cucumber plants grown under greenhouse conditions.

Treatments	Fruit Length (cm)	Fruit Diameter (cm)	No. of Fruit per Plant	Fruit Fresh Weight (g)	Fruit Firmness (Lbs. Inch^−2^)
T1	11.83 ^ab^	2.10 ^b^	10.00 ^cd^	735.68 ^b^	4.92 ^a^
T2	11.50 ^b^	2.10 ^b^	8.33 ^d^	512.66 ^c^	4.83 ^a^
T3	13.83 ^ab^	2.80 ^a^	13.33 ^a^	810.83 ^ab^	5.00 ^a^
T4	14.33 ^a^	2.83 ^a^	13.83 ^a^	920.00 ^a^	4.90 ^a^
T5	13.16 ^ab^	2.75 ^a^	13.16 ^ab^	884.50 ^ab^	5.00 ^a^
T6	13.16 ^ab^	2.43 ^ab^	12.66 ^ab^	782.50 ^ab^	4.83 ^a^
T7	13.33 ^ab^	2.58 ^a^	11.00 ^bc^	786.00 ^ab^	4.83 ^a^

Values followed by the same letter (s) within each column did not significantly differ according to the Duncan multiple comparison test at the 5% level. Each value is the average of 3 replicates. ± Values indicated to standard error (±SE). Normal phosphate (T1), rock phosphate (RP) (T2), RP + strain HM3 (T3), RP + strain HM3 (T4), RP + strain HM8 (T5), RP + strain HM10 (T6) and RP + mixture of the four strains (T7). ^a,b,c,d^: Values not sharing similar letters are significantly different (*p* > 0.05).

**Table 4:** Effect of chemical, organic, and biofertilizer treatments on soil and plant phosphorus concentration.

Treatments	Soil P (mg/kg)			Plant P (%)
	Before Planting	21 DAT	At Harvest	
T1	2.06 ^c^	12.52 ^f^	17.92 ^d^	0.43 ^c^
T2	1.16 ^e^	18.34 ^c^	13.90 ^g^	0.52 ^c^
T3	5.20 ^a^	18.50 ^c^	15.02 ^f^	0.52 ^c^
T4	2.48 ^b^	30.76 ^a^	26.36 ^a^	0.91 ^a^
T5	1.76 ^cd^	27.76 ^b^	22.40 ^b^	0.75 ^b^
T6	1.44 ^de^	17.42 ^d^	18.50 ^c^	0.55 ^c^
T7	1.51 ^de^	13.75 ^e^	16.08 ^e^	0.53 ^c^

Values followed by the same letter (s) within each column did not significantly differ according to the Duncan multiple comparison test at the 5% level. Each value is the average of 3 replicates. ± Values indicated to standard error (±SE). Normal phosphate (T1), rock phosphate (RP) (T2), RP + strain HM3 (T3), RP + strain HM3 (T4), RP + strain HM8 (T5), RP + strain HM10 (T6) and RP + mixture of the four strains (T7). FW = fresh weight and DW = Dry weight. (DAT) Days after transplanting. ^a,b,c,d,e,f,g^: Values not sharing similar letters are significantly different (*p* > 0.05).

**Table 5:** Physical and Chemical Properties of Soil and Water used for the Study.

Properties	Value	
	Soil	Water
Physical properties		
Sand (%)	94.1	-
Silt (%)	3.6	-
Clay (%)	2.3	-
Texture	Sand	-
Chemical properties		
^1^ pH	7.84	7.17
^2^ EC (dS·m^−1^)	0.508	1.55
^3^ Nutrient (ppm)		
Total N	168	-
Available P	0.450	-
Available K	45.0	40.0
^4^ Dissolved Ions (meq·L^−1^)		
(1) Dissolved anions (meq·L^−1^)		
Cl^−^	1.0	12.0
HCO_3_^−1^ + CO_3_^−2^	3.0	3.0
(2) Dissolved cations (meq·L^−1^)		
Na^+^	1.3	11.9
Ca^++^	3.0	4.0
Mg^++^	1.0	3.0

^1−^ pH was measured in the soil suspension (soil: water) at a ratio of (1:2.5) using a pH meter type (Jenway, model 3310) according to what was mentioned in the method of Page [104]. ^2−^ EC (dS m^−1^) was measured in the saturated soil paste extract using an EC-meter type (ELE, model 470) according to the method recommended in Jackson [105]. ^3−^ Total N was determined by the Kjeldahl method; P was extracted using the Olsen method; K by 1 N NH4OAc at pH 7 Page, [104]. ^4−^ The dissolved salts (cations + anions) were measured in the extract of saturated soil paste and determined using the EDTA solution titration method for calcium, magnesium, carbonate, and bicarbonate by titration with hydrochloric acid, and chloride by titration with silver nitrate, while, sodium was determined using a flame photometer according to the method mentioned by Jackson [105].

## Data Availability

Raw data is available upon request.
